# Supplementary material for: Legionella pneumophila Presence in Dental Unit Waterlines: A Cultural and Molecular Investigation in the West Bank, Palestine
Source: Trop Med Infect Dis. 2023 Oct 30;8(11):490. doi: 10.3390/tropicalmed8110490 (PMC10675536; doi:10.3390/tropicalmed8110490)
Supplement: Supplementary file 1 [file tropicalmed-08-00490-s001.zip › tropicalmed-2601904-supplementary.pdf]

Supplementary materials

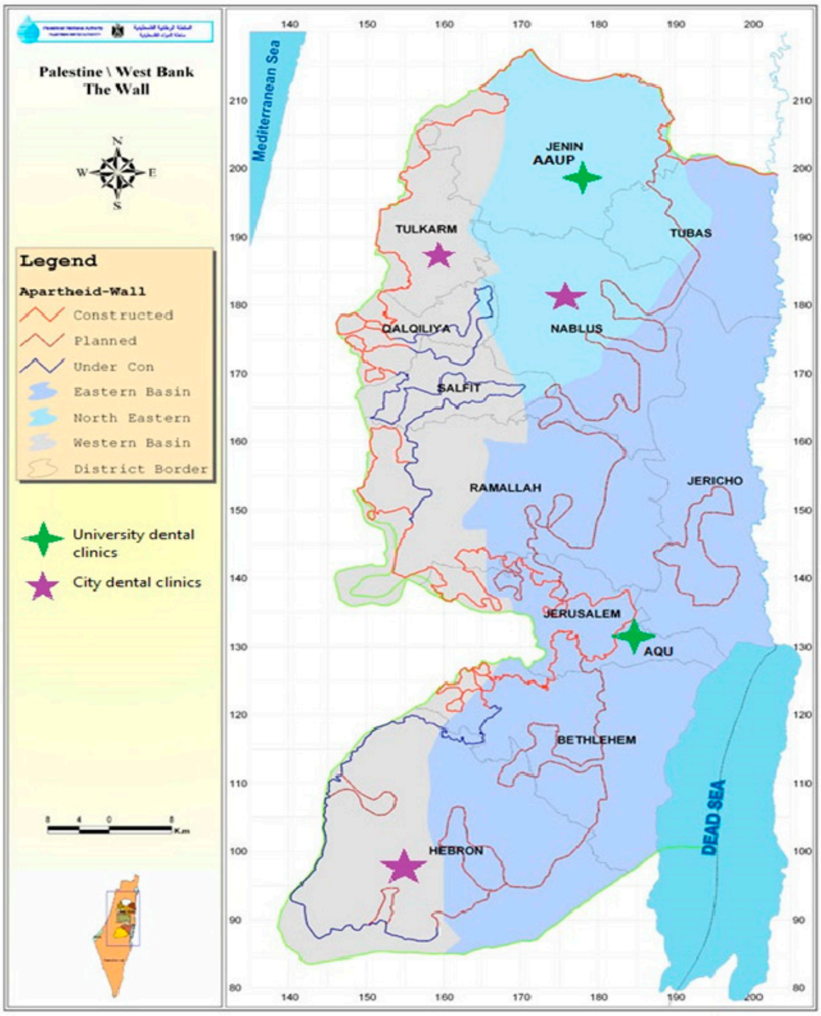

Figure S1: Sampling sites in the West Bank; Al-Quds University (AQU), Faculty of Dentistry in Abu Deis, East Jerusalem, and Arab American University in Jenin (AAUP), Faculty of Dentistry, and Dentists clinics in Nablus, Tulkarem, and Hebron. (Map adapted from Palestinian water authority PWA 2019).

Table S1. Sequence identity of 16S rRNA sequenced isolates

Isolate name	Accession No.	Database match with accession no.in parentheses	%Identity
AQU1_TW_Ps	SAMN36886996	*Legionella pneumophila* strain A194 chromosome (CP114578.1)	97.91
AQU2_DUWL_Ps	SAMN36886997	*Legionella pneumophila* 130b chromosome, complete genome (CP115860.1)	94.54
AQU3_TB_Ps	SAMN36886998	*Legionella pneumophila* strain A194 chromosome (CP114578.1)	95.46
AQU4_DUB_Ps	SAMN36886999	*Legionella pneumophila* strain SG1-QSBC08 chromosome (CP115861.1)	94.98
N1_TW_Ps	SAMN36887000	*Legionella pneumophila* subsp. pneumophila str. Philadelphia 1 (AE017354.1)	97.03
N2_TB_Ps	SAMN36887001	*Legionella pneumophila* strain OLDA, complete genome (CP016030.2)	96.83
N3_DUB_Ps	SAMN36887002	*Legionella pneumophila* strain A194 chromosome (CP114578.1)	98.16
T1_TW_Ps	SAMN36887003	*Legionella pneumophila* str. Paris complete genome (CR628336.1)	98.15
T2_DUWL_Ps	SAMN36887004	*Legionella pneumophila* 130b chromosome, complete genome (CP115860.1)	97.99
T3_TB_Ps	SAMN36887005	*Legionella pneumophila* str. Paris complete genome (CR628336.1)	97.06
T4_DUB_Ps	SAMN36887006	*Legionella pneumophila* 130b chromosome, complete genome (CP115860.1)	97.82
G1_TW_Ps	SAMN36887007	*Legionella pneumophila* str. Paris complete genome (CR628336.1)	98.26
G2_DUWL_Ps	SAMN36887008	*Legionella pneumophila* strain A194 chromosome (CP114578.1)	98.27
G3_TB_Ps	SAMN36887009	*Legionella pneumophila* strain AUSMDU00010536 isolate chromosome (CP045974.1)	97.35
G4_DUB_Ps	SAMN36887010	*Legionella pneumophila* strain A194 chromosome (CP114578.1)	96.12
